# Bilateral spontaneous retroperitoneal bleeding in a patient on nimesulide: a case report

**DOI:** 10.1186/1752-1947-5-568

**Published:** 2011-12-09

**Authors:** Iraklis C Mitsogiannis, Eleftherios Chatzidarellis, Andreas Skolarikos, Athanasios Papatsoris, Georgia Anagnostopoulou, Evangelos Karagiotis

**Affiliations:** 1 2nd Department of Urology, University of Athens, School of Medicine, Athens, Greece

## Abstract

**Introduction:**

Spontaneous retroperitoneal bleeding is a rare but potentially life-threatening event of varied etiology. Herein we report a case of bilateral non-traumatic retroperitoneal hemorrhage.

**Case presentation:**

A 50-year-old Greek man, who was on a non-steroidal anti-inflammatory agent (nimesulide) for ankylosing spondylitis, presented with a right retroperitoneal hematoma combined with contralateral subcapsular renal hematoma. Bleeding on his right side was successfully controlled by arterial embolization with coils, whereas the left renal hematoma was treated conservatively. His recovery period was uneventful.

**Conclusion:**

This is the first reported case of bilateral retroperitoneal bleeding in a patient receiving nimesulide for ankylosing spondylitis. The application of minimally invasive techniques resulted in the desired positive outcome with preservation of both renal units.

## Introduction

Spontaneous retroperitoneal bleeding is a rare event which can potentially be life-threatening. The majority of cases are due to ruptured renal lesions (such as angiomyolipomas); however, vascular disorders and anticoagulation therapy may also be the underlying cause. Presenting symptoms and clinical signs depend on the degree and duration of bleeding and therefore may vary significantly. We report a case of spontaneous bilateral retroperitoneal hematoma in a patient on nimesulide for ankylosing spondylitis.

## Case presentation

**Figure 1:**
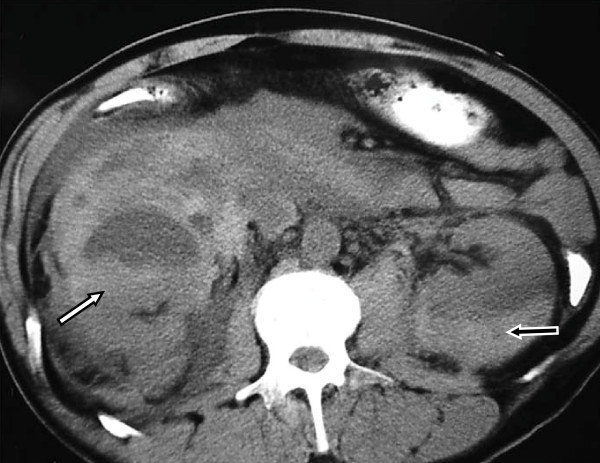
**Computed tomography scan demonstrating an extended right retroperitoneal hematoma (white arrow) combined with a left subcapsular renal hematoma (black arrow)**.

A 50-year-old Greek man presented at our Accidents and Emergency Department with acute right flank pain and gross hematuria of three-hour duration. Our patient reported no trauma, renal disease or coagulation disorder but had a history of ankylosing spondylitis for which he had received various courses of methylprednisolone and non-steroidal anti-inflammatory agents (NSAIDs) in the past; at the time of presentation our patient was on nimesulide, a selective cyclooxygenase-2 (COX-2) inhibitor. A clinical examination disclosed abdominal tenderness on the right, tachypnoea, low arterial pressure and tachycardia. A laboratory evaluation revealed severe anemia (hematocrit, 15%). A computerized tomography (CT) scan of his chest and abdomen demonstrated an extensive right retroperitoneal hematoma combined with a subcapsular hematoma of the left kidney (Figure 1). Atelectasis of his right lower lung lobe, bilateral pleural effusion and scattered lung infiltrations were also present.

**Figure 2:**
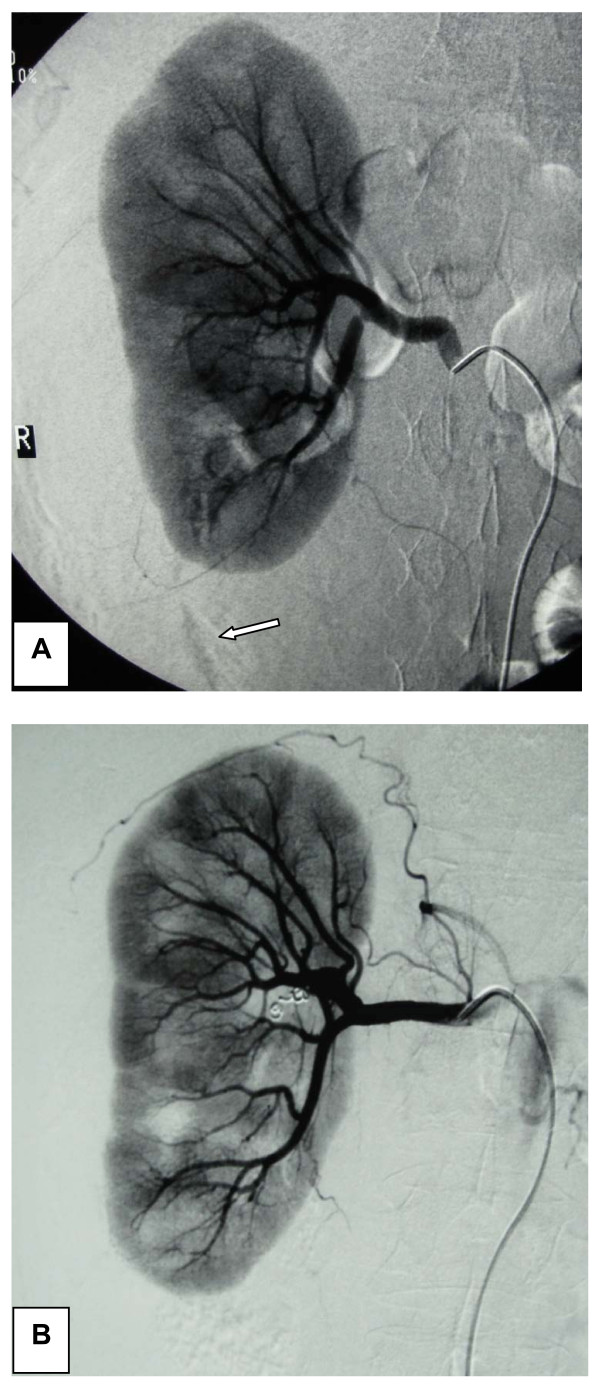
**Digital subtractive angiography**. **(A) **Angiography disclosed a bleeding low renal pole vessel on the right (arrow). **(B) **Occlusion of the bleeding vessel using coils.

Following initial resuscitation and stabilization, a digital subtractive angiography was carried out to accurately define the bleeding sites; a ruptured arterial branch of the lower pole of his right kidney was recognized, constantly bleeding at a low flow rate (Figure 2a). No specific bleeding site was recognized on his left kidney. The ruptured vessel was effectively occluded by selective embolization using coils (Figure 2b). Our patient recovered totally within two days.

**Figure 3:**
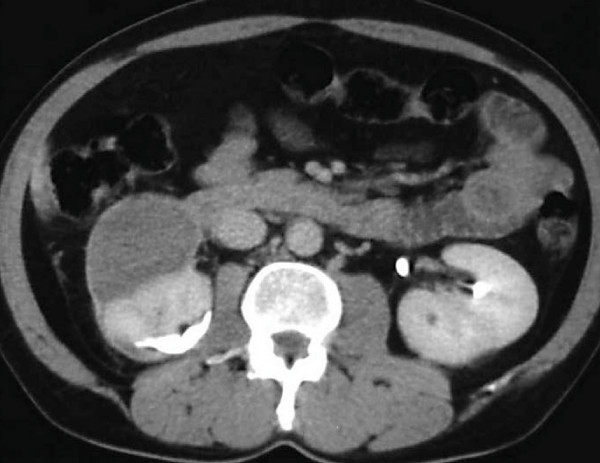
**The six-month follow-up computed tomography scan shows total elimination of the left subcapsular hematoma and a significant reduction of the retroperitoneal hematoma on the right**.

A repeat CT scan carried out 10 days later showed improvement of the thoracic findings and stabilization of the hematomas. Our patient was discharged home three weeks after the embolization and was followed by regular ultrasonography scans and laboratory tests (weekly during the first month and monthly thereafter). At the six-month follow-up, a CT scan revealed significant reduction of the right retroperitoneal hematoma and total absorption of the left subcapsular hematoma (Figure 3).

## Discussion

Spontaneous retroperitoneal hemorrhage is rarely reported in the literature and is predominantly due to the rupture of renal lesions, including angiomyolipomas and renal cell carcinomas [1]. Renal tumors account for 58% to 79% of cases of non-traumatic retroperitoneal hemorrhage [1,2]. Other causes of this potentially life-threatening entity are anticoagulation therapy [3,4] and vascular disorders, including aneurysms associated with autoimmune diseases, mainly polyarteritis nodosa [5,6].

Ankylosing spondylitis is a chronic inflammatory rheumatic disease characterized by significant pathological changes in the vertebral column (osteoporosis, ligament ossification or vertebral joint fusion), which eventually becomes unstable and therefore susceptible to fractures, spinal cord injury and epidural hematomas [7]. Amongst the treatment options, aspirin and other NSAIDs are commonly used to reduce pain and inflammation via inhibition of prostaglandin synthesis. These agents carry the risk of severe adverse events, primarily involving the digestive system (such as ulceration or bleeding). Nimesulide has previously been involved in a case of retroperitoneal hematoma in a patient receiving sertraline for depression [8]. To the best of our knowledge, this is the first reported case of spontaneous bilateral retroperitoneal bleeding in a patient on NSAIDs for ankylosing spondylitis.

Symptomatology of spontaneous retroperitoneal hematoma may vary significantly, from slight flank and/or abdominal pain to cardiovascular collapse, depending on the degree and duration of bleeding. In our patient, the symptoms and signs were clearly indicative of a severe hemorrhage necessitating emergency management. In less severe cases, the clinical presentation may not be so characteristic and the diagnosis may be delayed. A contrast-enhanced CT scan is regarded as the most sensitive diagnostic modality in revealing the bleeding site and identifying any underlying pathology [9]. In our case, no such pathology was shown on CT but the bilateral location of the hematomas was clearly demonstrated. Nonetheless, the underlying cause of a retroperitoneal hematoma may not be readily demonstrated during the acute phase of blood accumulation [9]; serial CT scans during the follow-up period may prove to be extremely valuable in unmasking renal lesions. Ultrasonography is less sensitive than CT in detecting renal pathology but may be used as an alternative for following up conservatively-treated retroperitoneal hematomas, as it is a rapid, easily available, cost-effective and radiation-free modality.

Angiography may have both a diagnostic and a therapeutic role in patients with retroperitoneal hemorrhage. Apart from delineating any ruptured vessels, it allows for selective arterial embolization during the same procedure. This approach obviates the need for invasive surgery and should always be considered as an option, especially when both kidneys are involved, as in our case. Surgical exploration is reserved for patients with uncontrolled bleeding or those with renal ischemia and non-viable renal parenchyma, although some authors advocate surgery in all cases with spontaneous rupture of the kidneys due to the high incidence of underlying malignancies [10]. Such an approach, however, might lead to a number of unnecessary nephrectomies.

In this case, a direct causative link between the use of nimesulide and the occurrence of the retroperitoneal bleeding cannot be clearly established. Regardless of the etiology, however, the selection of a minimally invasive approach, like arterial embolization, resulted in preservation of both renal units and an uneventful recovery.

## Conclusion

To the best of the authors' knowledge, this is the first reported case of bilateral spontaneous retroperitoneal hemorrhage in a patient on nimesulide for ankylosing spondylitis. Early diagnosis and prompt treatment are the mainstays of successful management of this rare untoward event. This case emphasizes the role of minimally invasive techniques, such as arterial embolization, in achieving a positive outcome.

## Consent

Written informed consent was obtained from the patient for the publication of this case report and any accompanying images. A copy of the written consent is available for review by the Editor-in-Chief of this journal.

## Competing interests

The authors declare that they have no competing interests.

## Authors' contributions

ICM and EC were the major contributors in writing the manuscript. AS and GA collected the data and helped in drafting the manuscript. AP and EK diagnosed, managed and followed-up the patient and searched the literature. All authors read and approved the final manuscript.
